# The Epidemiology and Clinical Features of Lymphangioleiomyomatosis (LAM): A Descriptive Study of 33 Case Reports

**DOI:** 10.7759/cureus.43513

**Published:** 2023-08-15

**Authors:** Jaini M Shah, Jaimin T Patel, Hriday Shah, Harika Dadigiri, Arya Alla, Pramil Cheriyath

**Affiliations:** 1 Internal Medicine, GCS Medical College Hospital and Research Centre, Ahmedabad, IND; 2 Internal Medicine, California Institute of Behavioral Neurosciences & Psychology, Fairfield, USA; 3 Internal Medicine, Saint Clare's Hospital, New Jersey, USA; 4 Internal Medicine, Hackensack Meridian - Ocean Medical Center, Brick, USA

**Keywords:** cystic lung changes, hmb-45, recurrent pneumothorax, pneumothorax, sporadic lymphangioleiomyomatosis

## Abstract

Lymphangioleiomyomatosis (LAM) is a rare, slow-growing metastasizing neoplasm in which smooth muscle-like cells infiltrate the lung parenchyma and cause cystic lung damage. The common early symptoms include shortness of breath, pneumothorax, and chest pain. Lymphangioleiomyomatosis mainly involves the lungs, kidneys, and lymph nodes. This study reviews the characteristics of lymphangioleiomyomatosis to identify any possible changes in the prevalence of symptoms of the disease.

We conducted a literature review of case reports on lymphangioleiomyomatosis from PubMed and Google Scholar. Variables of interest were age, gender, symptoms, vitals, immunostaining, and radiological findings. Data were transferred to an Excel spreadsheet (Microsoft Corporation, Redmond, WA), and mean, median, standard deviation, frequencies, and proportions were calculated using R version 1.1.456 (RStudio: Integrated Development for R. RStudio, PBC, Boston, MA). Lymphangioleiomyomatosis is a rare case and so not much of the literature could be found online. Thirty-three case reports were included in this study, and females accounted for 78.78% of the presentations. The average age was 38 years, SD 14.41 years. Shortness of breath was the most frequent symptom (60.6%), followed by pneumothorax (57.57%), chest pain (42.42%), cough (24.24%), and pleural effusion (1.25%).

## Introduction and background

Lymphangioleiomyomatosis (LAM) is a rare, slow-progressing, low-grade, metastasizing neoplasm. The invading cell in LAM develops from an unknown source and carries mutations in the tuberous sclerosis complex (TSC) genes that cause constitutive activation of the mechanistic target of rapamycin (mTOR) pathway, disrupted cellular proliferation, and a program of unsuccessful lymphangiogenesis due to which there is a characteristic aberrant smooth muscle-like cells infiltrating the lung parenchyma and causing cystic lung damage. [1]. It can also include extrapulmonary features consisting of renal angiomyolipomas and lymphatic involvement, e.g., lymphangioleiomyomas, and chylous effusions. There are two forms of LAM described - the sporadic form and the inherited form [2]. The prevalence of sporadic lymphangioleiomyomatosis( S-LAM ) is unknown but one study depicted that the sporadic form affects one in 400,000 adult women. In contrast to S-LAM, which primarily affects women, Tuberous sclerosis complex lymphangioleiomyomatosis (TSC-LAM) affects both men and women who have TSC. Cystic lung disease is thought to affect 10-30% percent of men with TSC [3]. The characteristics of lymphangioleiomyomatosis were analyzed in this study and the findings can help paint the typical picture of what LAM looks like and what physicians should expect when dealing with the disease.

## Review

Methods

Eligibility Criteria

All case reports that were published on the Internet under the name lymphangioleiomyomatosis.

Search Strategies

We performed a systematic search of lymphangioleiomyomatosis case reports utilizing Google, Medscape, Scopes, and scientific databases such as Google Scholar and PubMed

Data Collection Process and Data Items

Using standardized data extraction forms, data were extracted independently by two authors. We collected characteristics like age, gender, initial symptom presentation, vital signs, diagnostic imaging, immunostaining, and results on an Excel sheet (Microsoft Corporation, Redmond, WA), and these variables were analyzed.

Statistical Analysis

Patient demographic characteristics, disease manifestations, and causes were summarized descriptively and analyzed using R version 1.1.456 (RStudio: Integrated Development for R. RStudio, PBC, Boston, MA).

The methods of study and formats were followed from a previously published article [4].

Results

**Figure 1:**
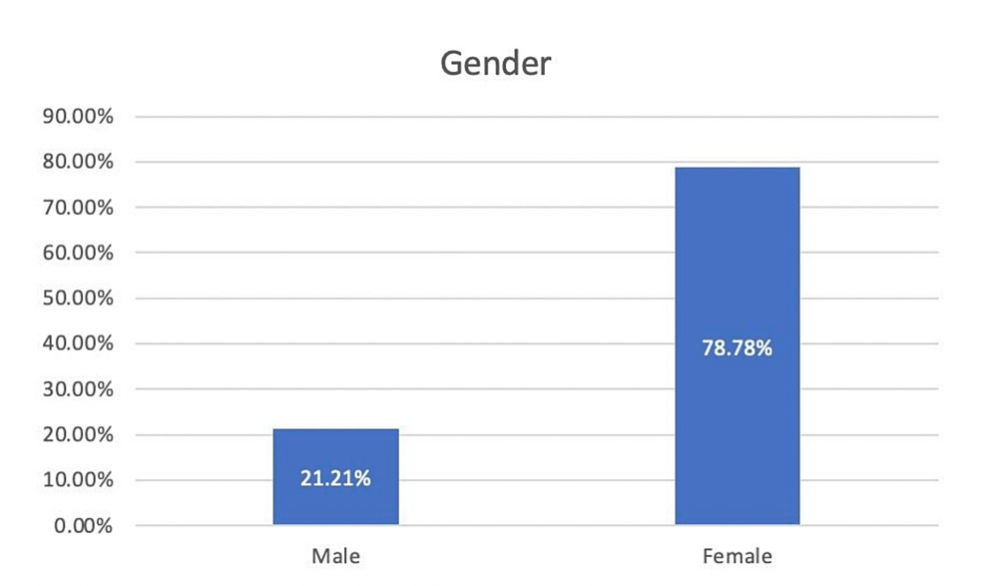
Percentage of affected males vs females

**Figure 2:**
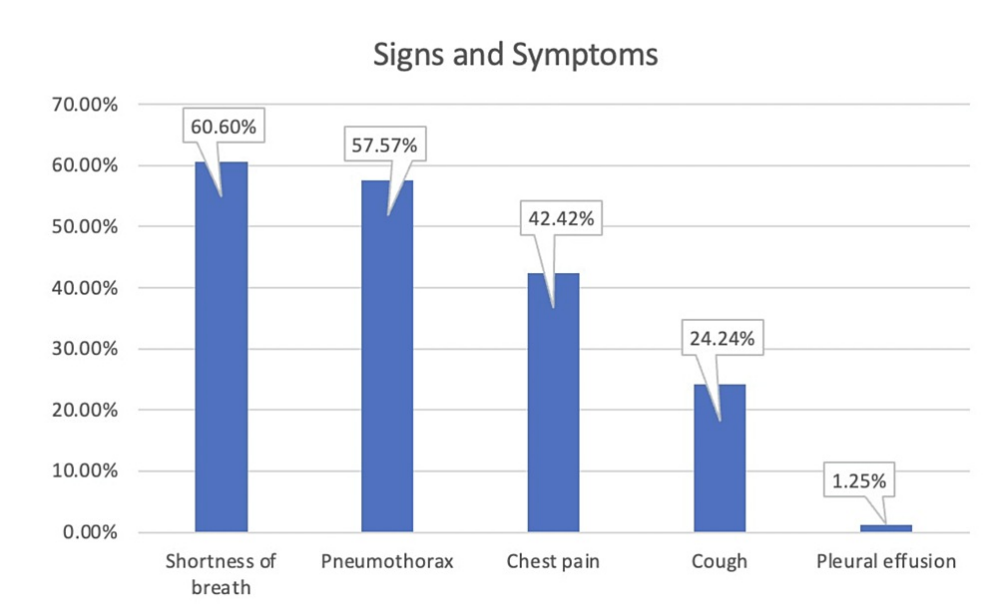
Initial clinical features of lymphangioleiomyomatosis

**Figure 3:**
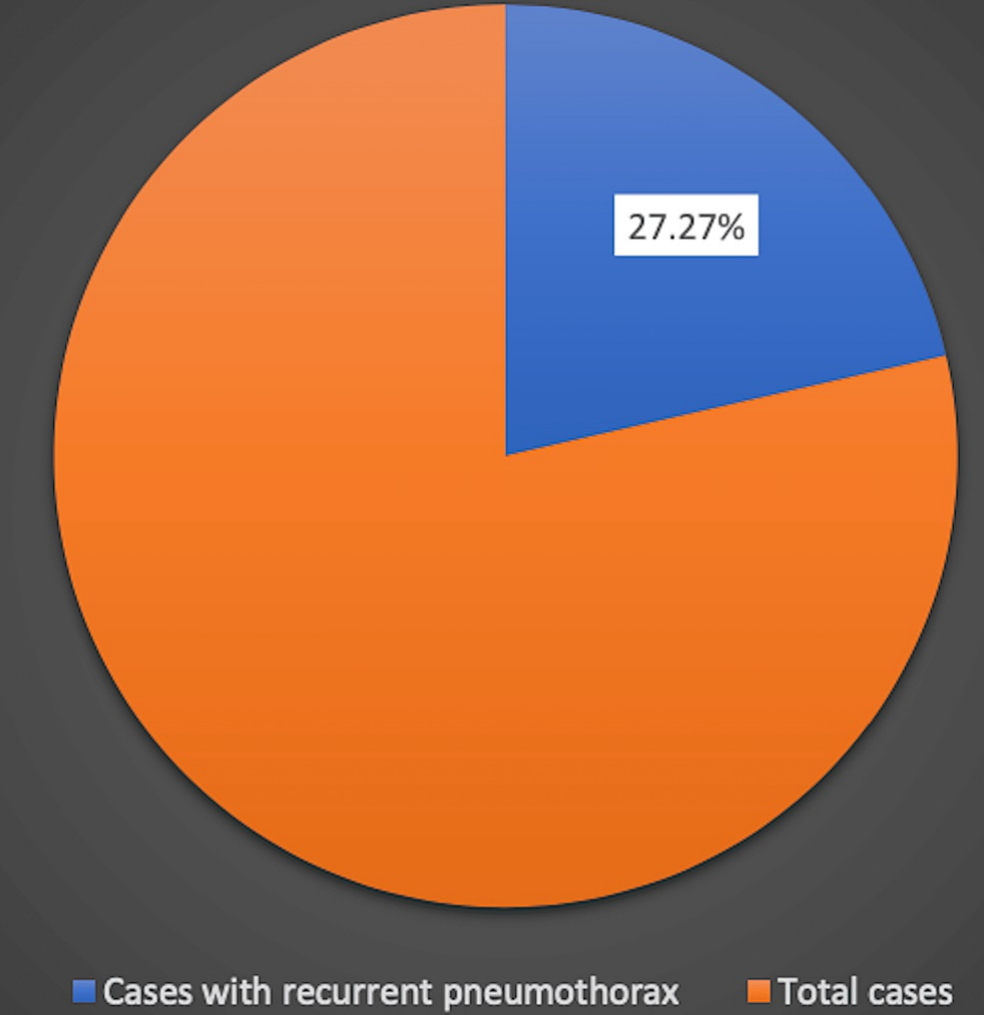
Patients who had recurrent episodes of pneumothorax

We reviewed 33 case reports available online and gathered all the data regarding the age, gender, characteristics, and presentation of initial symptoms. Table 1 describes the proportion of initial clinical presentations of lymphangioleiomyomatosis. The average age of the population was 38 years, with a standard deviation of 14.41 years. Females accounted for 78.78% of the cases while males accounted for 21.21% (Figure 1). The most common symptom of lymphangioleiomyomatosis was shortness of breath (60.6%%), followed by pneumothorax (57.57%), chest pain (42.42%), and cough (24.24%) (Figure 2, Figure 3). Pleural effusion was noted in 1.25% of cases. Out of all the collected data, 14 cases underwent video-assisted thoracoscopic surgery (VATS). Thirty-one cases were reported to have positive immunostaining for human melanoma black 45 (HMB-45). Twenty-seven cases showed cystic changes in the CT scan. Ten cases showed extrapulmonary involvement most being renal angiomyolipomas. Recurrent pneumothorax was observed in 27.27% of cases.

**Table 1:** Characteristics and presentation of initial signs and symptoms, diagnostic imaging, and immunostaining (n = 33) VATS - video-assisted thoracoscopic surgery, HMB-45 - human melanoma black 45, FEV1 - forced expiratory volume in the first second

Parameter	Value(±SD)
Age	38 (±14.41)
Female	78.78%
Male	21.21%
Shortness of breath	60.6%
Pneumothorax	57.57%
Chest pain	42.42%
Cough	24.24%
Pleural effusion	1.25%
Recurrent Pneumothorax	27.27%
Extrapulmonary involvement	30.30%
FEV1	
Decrease	66.67%
Normal	33.33%
CT scan - Cystic changes in lungs	81.25%
VATS performed	42.42%
Immunostaining for HMB-45 positive	94.11%

Discussion

Lymphangioleiomyomatosis (LAM) is a rare neoplasm that is characterized by the infiltration of aberrant smooth muscle-like cells in the lung parenchyma, which results in cystic lung damage [1]. It primarily affects women and is also associated with lymphatic abnormalities and abdominal tumors [5].

The exact cause of LAM is unknown, although there is a hypothesis suggesting a potential role of female sex hormones in its pathogenesis. LAM can occur spontaneously and shares similarities with tuberous sclerosis (TS), as some TS patients also experience LAM, leading to the hypothesis that LAM may be a form of TS [6]. Two forms of LAM have been identified. The sporadic form is traditionally associated with mutations in the TSC2 gene, although not all sporadic LAM patients have these mutations. Other gene mutations, including TSC1 and other potential causal genes, are being investigated, but their role as drivers in LAM remains unclear. In LAM, larger and more frequent angiomyolipomas are often linked to TSC2 mutations and present a slightly distinct phenotype compared to TSC1- and TSC2-related diseases in tuberous sclerosis. The protein products of TSC1 and TSC2, hamartin, and tuberin, respectively, form a complex that inhibits the activity of the protein mTOR, regulating cellular proliferation. In LAM, the deletion of hamartin or tuberin results in constitutive activation of mTOR and cellular proliferation [7].

LAM exhibits a wide range of clinical symptoms. The most common respiratory symptoms in our study at presentation included shortness of breath, pneumothorax, and chest pain. Extrapulmonary presentations can include intraabdominal bleeding or the discovery of abdominal tumors caused by lymphadenopathy, lymphangioleiomyoma, or angiomyolipoma. Lymphangioleiomyomas, cystic tumors that usually develop in the retroperitoneum, pelvis, and abdomen, may cause specific symptoms such as fluctuating size, nausea, stomach pain, edema in the extremities, urinary complaints, or an acute abdomen. Angiomyolipomas are benign tumors that mainly affect the kidneys, often asymptomatic, but can lead to bleeding when they grow larger [8].

Diagnosing LAM can be challenging, as its symptoms may overlap with other respiratory diseases. Conventional chest X-rays may show non-specific indications of pneumothorax or pleural effusions. High-resolution computed tomography (HRCT) is highly sensitive and specific for detecting LAM and can reveal the hallmark diagnostic finding of numerous thin-walled pulmonary cysts (81.25%). Cysts formed in the lungs can be due to smooth muscle cell proliferation, which can damage the healthy tissue and form fluid-filled pockets preventing air from moving in and out of the lungs. Other radiographic findings may include reticulonodular opacities, pleural thickening, hyperinflation, and focal ground-glass opacities can be noticed as LAM advances. Elevated levels of vascular endothelial growth factor-D (VEGF-D) in the blood can help confirm the diagnosis of LAM without requiring a lung biopsy. However, in some cases, a lung biopsy may be necessary, which can be performed using transbronchial biopsy or video-assisted thoracoscopic lung biopsy. Although transbronchial biopsy is less invasive and can be done as an outpatient procedure, VATS is preferred because more amount of lung tissue can be obtained [3,9].

Histologically, LAM lesions consist of two major cell morphologies: small spindle-shaped cells and cuboidal epithelioid cells. These cells stain positive for smooth muscle actin, vimentin, desmin, estrogen and progesterone receptors, and the monoclonal antibody human melanoma black (HMB-45). HMB-45 staining is particularly useful in distinguishing LAM from other lung lesions with smooth muscle predominance [10].

In some cases, hormonal therapies like progesterone or hormonal contraceptives may be considered, as hormone fluctuations can influence the progression of LAM. However, the effectiveness of hormonal therapy is still under investigation, and more research is needed in this area. The management of LAM includes lifestyle modifications such as weight reduction. Lung transplantation can be considered definitive management for LAM. Predicting the prognosis of this disease for individual patients can be difficult. However, the histopathological extent of the disease and some lung function variables like FEV1 are found to have some prognostic value. Due to the increased risk of pneumothorax, patients with LAM are often advised against air travel. Pulmonary rehabilitation and progesterone therapy have shown some beneficial effects in certain cases. The mainstay of medical therapy for LAM involves the use of mTOR (mammalian target of rapamycin) inhibitors. These drugs, such as sirolimus (rapamycin) and everolimus, work by inhibiting the mTOR pathway, which plays a role in cell proliferation and growth. By suppressing the abnormal growth of smooth muscle cells in the lungs, mTOR inhibitors can help slow down the progression of LAM and reduce symptoms such as shortness of breath and lung function decline [10,11].

After excluding duplication, we have included published articles in our study, which consists of 33 case reports (Table 2) [12-42].

**Table 2:** References of case reports and case studies included in this study

No	Author	Study name	Brief
1	Kania BE et al. [12]	Lymphangioleiomyomatosis: A Case Report and Review of Clinical Features and Management	A 39-year-old female presented to the emergency department with a chief complaint of two days of sharp, left-sided chest pain radiating to her left neck and left upper back associated with shortness of breath and an episode of presyncope.
2	Yamazaki A et al. [13]	An early case of pulmonary lymphangioleiomyomatosis diagnosed by video-assisted thoracoscopic surgery	A 28-year-old woman presented to a local clinic with chest pain and her chest X-ray film showed left pneumothorax.
3	Sathirareuangchai S et al. [14]	Pulmonary Lymphangioleiomyomatosis: A Case Report and Literature Review	The patient is a 41-year-old woman with a medical history of hypertension and dyslipidemia. She presented to the emergency department at another institution with sudden shortness of breath and left-sided pleuritic chest pain.
4	Cong CV et al. [15]	Pulmonary lymphangioleiomyomatosis (LAM): A literature overview and case report	A 36-year-old female patient was admitted to the hospital due to chest pain. The disease had progressed 7 days before admission when the patient presented with right chest pain and shortness of breath and without fever or cough.
5	Rhee JA et al. [16]	Lymphangioleiomyomatosis: A Case Report and Review of Literature	A 55-year-old woman presented three days after a sudden onset of right-sided chest pain, pleuritic and positional in nature, associated with an acute onset of shortness of breath.
6	Zhou B et al. [17]	Pulmonary lymphangioleiomyomatosis in a 46-year-old female: A case report and review of the literature	A 46-year-old female in Northwestern China presented with exertional dyspnea, which occurred 1 month prior and progressed gradually. The patient did not report any fever, chills, cough, chest pain, or hemoptysis.
7	Albuquerque PR et al. [18]	Pulmonary Lymphangioleiomyomatosis: A Case Report	A 46-year-old woman with progressive dyspnea for 3 years.
8	Patel MV [19]	A Rare Case Report of Lymphangioleiomyomatosis	A 24-year-old female presented with complaints of gradually progressive breathlessness for 1 month.
9	Verma SK et al. [20]	PULMONARY LYMPHANGIOLEIOMYOMATOSIS (PLAM)	A 28-year-old female was admitted to our hospital with a complaint of dry cough, exertional dyspnoea, and chest pain for the last 2 years.
10	Nikmanesh Y et al. [21]	Sporadic Lymphangioleiomyomatosis Disease: A Case Report	A 31-year-old woman was admitted. She had been experiencing shortness of breath for several weeks.
11	Li CJ et al. [22]	Pulmonary lymphangioleiomyomatosis - a case report	A female patient, aged 23, complained of the activities of shortness of breath after 10 more than a month, adding to more than 1-week.
12	Kang HW et al. [23]	Pulmonary lymphangioleiomyomatosis in a male	A 22-year-old male presented to the hospital with complaints of dyspnea.
13	Aubry MC et al. [24]	Pulmonary Lymphangioleiomyomatosis in a Man	A 39-year-old African American man was seen at an emergency room after an epileptic seizure. He had a history of recurrent seizures, schizophrenia, and mild mental retardation. He also had a history of lung disease of undetermined nature.
14	Wakida K et al. [25]	Lymphangioleiomyomatosis in a Male	A 17-year-old male with a histopathologic diagnosis of lymphangioleiomyomatosis after surgery for pneumothorax.
15	Kabi A et al. [26]	A rare pulmonary lymphangioleiomyomatosis disease in a male with tuberous sclerosis complex	An 18-year-old boy presented to the emergency department with a history of seizures followed by loss of consciousness.
16	Baldi S et al. [27]	Pulmonary lymphangioleiomyomatosis in postmenopausal women: report of two cases and review of the literature	Case 1: A 59-year-old woman, who had no children, and was 8 yrs postmenopausal, was admitted to a hospital with an 8-month history of dyspnea with exertion. Case 2: A 62-year-old woman, with two children, complained of dyspnoea and right chest pain.
17	Liu Y et al. [28]	Lymphangioleiomyomatosis: a case report and review of diagnosis and treatment	A 66-year-old man was admitted with the chief complaint of cough with bloodstained phlegm for approximately 3 days.
18	Urban T et al. [29]	Pulmonary lymphangioleiomyomatosis. A study of 69 patients	Case 1: A 31-year-old woman was admitted with pulmonary lymphangioleiomyomatosis. She had been well until 36 months earlier, when a left nephrectomy was performed for renal angiomyolipoma. Thirteen months before inclusion, she was admitted to another institution for a first episode of right pneumothorax managed by a chest tube. Case 2: A 37-year-old woman was admitted with complete right pneumothorax, which was managed by a chest tube.
19	Han B et al. [30]	A second hit somatic (p.R905W) and a novel germline intron-mutation of TSC2 gene is found in intestinal lymphangioleiomyomatosis: a case report with literature review	A 9-year-old girl with a full-blown TSC presented with abdominal masses detected during a routine check-up.
20	Han JM et al. [31]	A case of lymphangioleiomyomatosis originated in the pelvic cavity	A 46-year-old nulligravida presented with complaints of vaginal bleeding for 15 days. Her past history was uneventful except for a left salpingectomy for a benign cyst at age 29.
21	Erginoz E et al. [32]	Leiomyomatosis-like lymphangioleiomyomatosis A case report of the colonic manifestation of tuberous sclerosis	A case of a 28-year-old woman presenting with symptoms of severe abdominal pain and nausea with a medical history of cardiac rhabdomyoma, adenoma sebaceum, Ash leaf spots, bilateral renal angiomyolipomas, and retinal hamartoma, which are manifestations of tuberous sclerosis complex.
22	Silva R et al. [33]	Pulmonary lymphangioleiomyomatosis: Report of one case	A 33-year-old woman consulted for chest pain and respiratory pressure.
23	Ağaçkiran Y et al. [34]	Pulmonary Lymphangioleiomyomatosis: A Rare Case	A 46-year-old female patient presented with cough, sputum persisting for a year, and dyspnea on exertion for three weeks.
24	Pan LH et al. [35]	Pulmonary Lymphangioleiomyomatosis: A Case Report with Immunohistochemical Details and DNA Analysis	A 47-year-old woman presented with pulmonary lymphangioleiomyomatosis (PLAM) involving the bilateral lung and slight pulmonary function abnormality.
25	Koc-Günel S. [36]	A Case of Lymphangioleiomyomatosis (LAM) of the Lung in a Patient with a History of Breast Cancer	A 47-year-old woman presented as an emergency with an exacerbation of a four-month history of shortness of breath and dry cough.
26	Denoo X et al. [37]	Successful Treatment of Pulmonary Lymphangioleiomyomatosis With Progestins	A 36-year-old patient was admitted to the emergency department with a dry cough, a morning fever, and diffuse sweating.
27	Yamanaka S et al. [38]	Two Kinds of Cystic Lung Lesions with Pulmonary Lymphangioleiomyomatosis in a Male	A 34-year-old male with frequent recurrence of right pneumothorax was admitted.
28	Han MK et al. [39]	Apparent Sporadic Lymphangioleiomyomatosis in a Man as a Result of Extreme Mosaicism for a TSC2 Mutation	A 48-year-old African-American man with a history of hypertension and diabetes presented to the emergency department with sudden onset of left-sided chest pain.
29	Sinclair W et al. [40]	Lymphangioleiomyomatosis presenting in a postmenopausal woman	A 72-year-old Caucasian woman was admitted to the hospital because of progressive respiratory failure.
30	Jain VV et al. [41]	Recurrent Pneumothorax in a Young Female with Pulmonary Lymphangiomyomatosis A Case Report and Overview of Literature	A 36-year-old female, non-smoker, presented to us with acute onset breathlessness and sudden-onset right-sided chest pain. Clinically she had tachypnea, tachycardia, and hyper-resonant percussion note with decreased breath sounds on the right side of the chest.
31	Alkemade L et al. [42]	Initial presentation of lymphangioleiomyomatosis in the third trimester of pregnancy	A 36-year-old primigravida presented with the main complaint of acute onset dyspnoea and worsening stabbing pain in the chest and left shoulder.

## Conclusions

The characteristics of lymphangioleiomyomatosis (LAM) were investigated in this study. Several symptoms and their corresponding values have been listed. Based on the review and analysis of all the case reports on lymphangioleiomyomatosis, we observed that lymphangioleiomyomatosis is most commonly seen in females (78.78%), with an average onset age of 24-52 years. The majority of patients present with a wide range of symptoms, from shortness of breath to pleural effusion and the associated symptoms. The main entity for diagnosis is imaging by CT scan, which depicts cystic changes in the lungs. Extrapulmonary manifestations like renal angiomyolipomas and lymphatic involvement, e.g., lymphangioleiomyomas and chylous effusions, can also occur. This paints a picture of the landscape of LAM and what physicians should expect when dealing with the disease.
